# Structure and Catalysis of Fe(III) and Cu(II) Microperoxidase-11 Interacting with the Positively Charged Interfaces of Lipids

**DOI:** 10.3390/molecules22081212

**Published:** 2017-07-26

**Authors:** Tatiana Prieto, Vinicius Santana, Adrianne M. M. Britto, Juliana C. Araujo-Chaves, Otaciro R. Nascimento, Iseli L. Nantes-Cardoso

**Affiliations:** 1 Universidade Federal do ABC, Santo André 09210-170, SP, Brazil; tati_prieto@yahoo.com (T.P.); adriannemmb@gmail.com (A.M.M.B.); casares.ju@gmail.com (J.C.A.-C.); 2 Universidade de São Paulo, Instituto de Física de São Carlos, São Carlos 13400-970, SP, Brazil; vstadeu@gmail.com

**Keywords:** microperoxidase-11, CTAB micelles, DODAB large vesicles, MCD, EPR

## Abstract

Numerous applications have been described for microperoxidases (MPs) such as in photoreceptors, sensing, drugs, and hydrogen evolution. The last application was obtained by replacing Fe(III), the native central metal, by cobalt ion and inspired part of the present study. Here, the Fe(III) of MP-11 was replaced by Cu(II) that is also a stable redox state in aerated medium, and the structure and activity of both MPs were modulated by the interaction with the positively charged interfaces of lipids. Comparative spectroscopic characterization of Fe(III) and Cu(II)MP-11 in the studied media demonstrated the presence of high and low spin species with axial distortion. The association of the Fe(III)MP-11 with CTAB and Cu(II)MP-11 with DODAB affected the colloidal stability of the surfactants that was recovered by heating. This result is consistent with hydrophobic interactions of MPs with DODAB vesicles and CTAB micelles. The hydrophobic interactions decreased the heme accessibility to substrates and the Fe(III) MP-11catalytic efficiency. Cu(II)MP-11 challenged by peroxides exhibited a cyclic Cu(II)/Cu(I) interconversion mechanism that is suggestive of a mimetic Cu/ZnSOD (superoxide dismutase) activity against peroxides. Hydrogen peroxide-activated Cu(II)MP-11 converted Amplex Red^®^ to dihydroresofurin. This study opens more possibilities for technological applications of MPs.

## 1. Introduction

**Figure 1:**
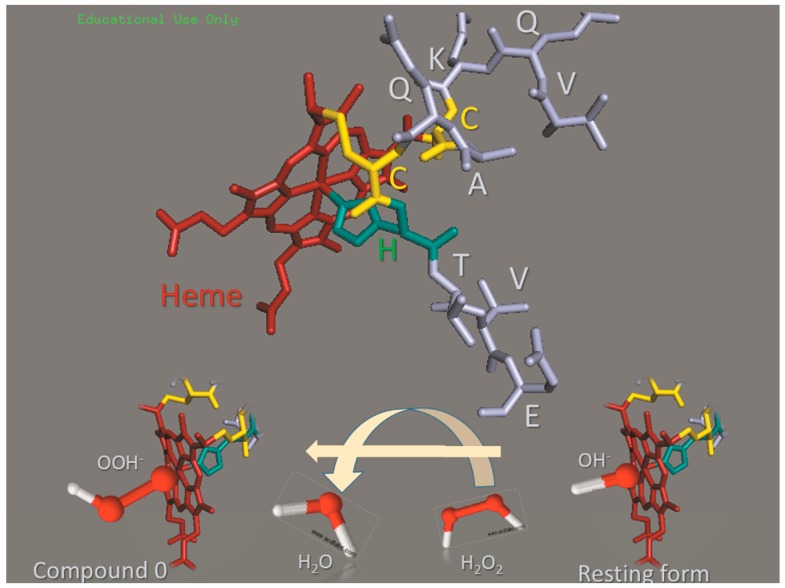
Cartoon of the microperoxidase structure. The upper side of the figure shows the MP-11 structure with the histidine side chain (blue) as a fifth axial ligand and the thioether bonds with cysteine residues (yellow) highlighted. The lower side of the figure represents the formation of Compound 0 by the replacement of water (OH^−^) by a hydrogen peroxide molecule (HO-O^−^) at the sixth axial position of heme iron. The microperoxidase structure was generated in the PyMol, 4.3 software by hiding part of Fe(III) horse heart cytochrome c structure available in Protein Data Bank (1HRC). Water and hydrogen peroxide molecules were designed using 3D representation in ChemSketch 2016.1 (free version for educational use only).

The properties of the heme iron when associated to peptides and proteins are modulated by the polypeptide chains folded around the prosthetic group. The polypeptide chain provides both a specific microenvironment and the axial ligands that determine the heme iron spin state and redox potential. Therefore, the iron protoporphyrin IX, the heme group, participates in a diversity of processes when associated with different polypeptide chains, such as oxygen transport in hemoglobin, hydrogen peroxide cleavage in catalase, electron transport in respiratory cytochromes and others. The tryptic digestion of respiratory cytochrome c converts this protein, evolutionarily tailored for electron transport, into peroxidases that are known as microperoxidases 6, 8, 9, and 11. Microperoxidases (MP) are numbered according to the number of amino acids in the remaining peptide chain [1,2]. Microperoxidases are also produced by biosynthetic routes via the production of recombinant proteins [3,4]. The peroxidase activity of MPs is favored by the lack of M80, the sixth ligand of cytochrome c heme iron. The tryptic fragment of MPs retains the heme group covalently attached to cysteine residues 14 and 17, the fifth coordination with H18 and lacks M80. In the case of MP-11, the peptide chain encompasses the sequence 11–21 of respiratory cytochrome c (Figure 1). Therefore, in an aqueous medium, a water molecule occupies the sixth coordination position of the resting form of Fe(III)MP-11. MP-11 and the other MPs exhibit peroxidase activity because water is easily displaced from the heme iron sixth coordination position by a peroxide molecule (Figure 1) [5,6,7,8]. The catalytic mechanism of microperoxidases is similar to that of the heme peroxidases, such as horseradish peroxidase. Thus, microperoxidases can reduce peroxides coupled to the oxidation of a variety of substrates [9]. The catalytic cycle of heme peroxidases involves the ligation of a peroxide molecule to heme iron leading to the formation of the first intermediate, Compound 0 (Porphyrin-Fe(III)-OOH). The second intermediate may be Compound I or Compound II. Compound I (Porphyrin^•+^-Fe^4+^ = O) is formed by the heterolytic cleavage of peroxides [10]. Compound II (porphyrin-Fe^4+^ = O) can be produced by one electron reduction of Compound I or directly from Compound 0 when the enzyme promotes homolytic cleavage of peroxides. The lack of a specific substrate-binding site enables the high valence states of MPs to catalyze the oxidation or hydroxylation of a wide variety of substrates, mimicking the peroxidases and cytochrome P-450, respectively.

A diversity of factors such as association to lipid bilayers [11], detergents [12], metallic nanoparticles [13], mesoporous silica [14,15,16], and the addition of endogenous and exogenous axial ligands [17,18,19] can modulate the catalytic mechanism of microperoxidases. Microperoxidases have been extensively used as a model for the study of peroxidase mechanisms. However, these studies conducted to the finding of numerous applications for these heme peptides such as photoreceptors, sensing, mainly related to the detection of hydrogen peroxide in vitro and in vivo [2,5,7,10,20,21,22,23,24,25,26,27,28], microzymes, drugs, and hydrogen evolution [29]. Interestingly, the latter application resulted from the replacement of the native central metal ion iron, by cobalt. The technological and therapeutic uses of microperoxidases are advantageous for several reasons. These microenzymes are water-soluble, biosynthetic, biodegradable, biocompatible and environmentally-friendly [30].

Considering the potential of metal-substituted microperoxidases, in the present study, we show the modulation of the activity of Fe(III)- and Cu(II)MPs by their association with the positively charged interfaces of lipids. Fe(III)- and Cu(II)MPs are oxidation states of the respective metal ions that are stable in an aerated medium.

## 2. Results

Microperoxidase-11 (MP-11) was converted to the free base form by the treatment with hydrogen fluoride. The free base MP-11 was incubated with CuSO_4_ to produce Cu-substituted MP-11. Both Fe(III) and Cu(II)MP-11 were associated with two types of pH-independent positively charged interfaces: CTAB micelles and DODAB liposomes. Metal substitution and association with cationic interfaces affect the physical chemistry properties of microperoxidase solutions.

### 2.1. Cu(II) and Fe(III)MP-11 in Homogeneous and Heterogeneous Media

**Figure 2:**
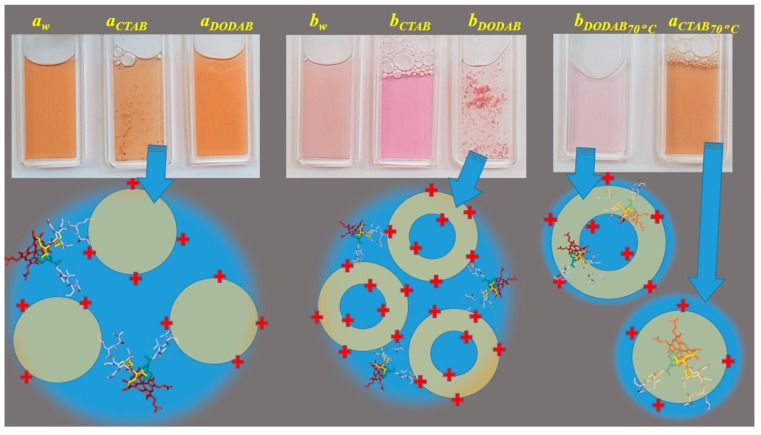
Snapshot of MP-11 solutions in homogeneous and heterogeneous media. Left and center panels show, respectively solutions of Fe(III) and Cu(II)MP-11. In each panel, each cuvette shows, respectively, MP-11 in HEPES-buffered aqueous solution, CTAB micelles, and DODAB liposomes at room temperature. Right panel shows, from left to right, the cuvettes containing, respectively Cu(II)MP-11 in DODAB and Fe(III)MP-11 in CTAB, after heating at 70 °C. The heating solubilized the aggregates that were shown in the left and center panels. Below the snapshots of cuvettes, the drawings represent possible MP-11/lipid structure interactions that are present in the flocculated systems at room temperature and the solubilized systems after heating. The drawings are not in scale.

Cu(II) and Fe(III)MP-11 in aqueous milieu resulted, respectively, in pale pink and red-brown solutions (a*_w_* and b*_w_*, respectively). The pink color of Cu(II)MP-11 solution was significantly intensified in the presence of CTAB micelles (b*_CTAB_*). CTAB micelles and DODAB LUVs promoted, at room temperature, flocculation of Fe(III)MP-11 and Cu(II)MP-11 solutions, respectively (Figure 2, a*_CTAB_* and b*_DODAB_*, respectively). DODAB LUVs did not promote flocculation of Fe(III)MP-11 at room temperature (Figure 2, a*_DODAB_*). The heating of the flocculated Fe(III)MP-11 in CTAB micelles, and Cu(II) MP-11 in DODAB LUVs at 70 °C promoted complete solubilization and stabilization of the systems that was not reversed by cooling (Figure 2, a*_CTAB70 C_* and b*_DODAB70 C_*, respectively). These results suggest that suspensions of MP-11 with CTAB micelles and DODAB are stabilized by a hydrophobic interaction of the heme peptide with the lipid structures. The interaction of the MP-11 with lipids is expected to be facilitated by a liquid crystalline phase promoted by heating. The hydrophobic interaction is in some way facilitated for Cu(II)MP-11 that did not require a liquid crystalline state of DODAB liposomes for stabilization. The interaction of Cu(II) and Fe(III)MP-11 with positively charged lipid supramolecular aggregates were also characterized by zeta potential and spectroscopic techniques of UV-visible, MCD and EPR to determine the structural effects of the association with cationic lipid assemblies.

Table 1 shows the effect of the ligation of Fe(III) and Cu(II)MP-11 on the zeta-potential (ξ) of CTAB micelles and DODAB vesicles. The binding of Fe(III) and Cu(II)MP-11 on CTAB micelles, and DODAB vesicles were corroborated by the change of the zeta potential to less positive values.

### 2.2. UV-Visible Spectral Features of Cu(II) and Fe(III)MP-11 in Homogeneous and Heterogeneous Media

**Figure 3:**
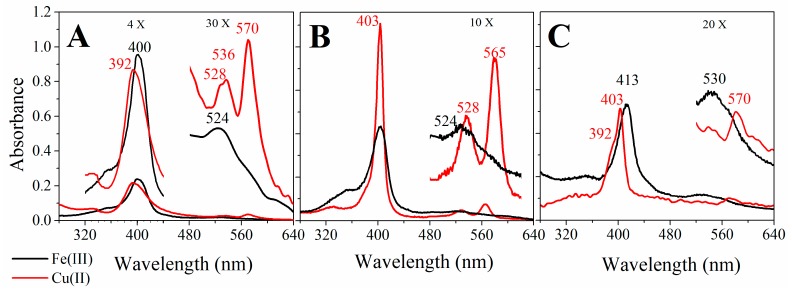
Comparative UV-visible spectra of Fe(III) or Cu(II)MP-11 in homogeneous and heterogeneous media. (**A**) The homogeneous medium is 5 mM HEPES buffer at pH 7.4. The heterogeneous medium is a suspension of (**B**) HEPES buffered 20 mM CTAB micelles and (**C**) HEPES buffered 2 mM DODAB LUVs. The spectra of 20 µM Fe(III) (black line) and Cu(II) (red line) MP-11 were recorded using a 0.1 cm quartz cuvette at 25 °C. For the systems with low colloidal stability, a 30 min of heating preceded the spectral analysis. The principal λ_max_ values are indicated in the spectra.

Figure 3A–C show the UV-visible spectra of Fe(III) and Cu(II)MP-11 in an aqueous milieu, CTAB micelles, and DODAB LUVs, respectively.

Figure 3A–C show the features of the electronic absorption spectra (280–640 nm) of Fe(III), and Cu(II)MP-11 (black and red lines, respectively) in HEPES buffered water, CTAB micelles, and DODAB LUVs, respectively. The association with CTAB micelles and DODAB LUVs, more significantly with the former lipid aggregate, promoted changes in the intensity and maximal λ of the porphyrins. The broader and low-intensity bands of Fe(III) and Cu(II)MP-11 in HEPES buffered water (Figure 3A) are consistent with aggregation of the heme peptides. The spectrum of Cu(II)MP-11 features like a composite spectrum with the contribution two population of heme peptides. The broad Soret band has the contribution of species peaking at 392, and ~403 nm and the Q_0-1_ band exhibits the contribution of a species peaking at 528 and 536 nm. Cu(II)MP-11 associated with CTAB micelles shows a narrow and high-intensity Soret band peaking at 403 nm and Q_0-1_ and Q_0-0_ bands peaking at 528 and 565 nm, respectively. In the presence of CTAB micelles the contribution of the species with Soret band at 392 nm and Q-band at 536 nm, probably a pentacoordinate Cu(II) heme peptide, is not present. Cu(II)MP-11 associated with CTAB micelles might be axially coordinated with the piperazine moiety of HEPES structure or with the terminal amino group of the peptide chain of another MP-11 molecule. Fe(III)MP-11 associated with CTAB micelles also exhibited Soret band peaking at 403 nm that is suggestive of the presence of hexacoordinate heme iron with a strong field ligand at the sixth coordination position. The spectral changes observed for MP-11 associated with CTAB micelles should also be promoted by the heme moiety buried in a hydrophobic environment. Cu(II)MP-11 associated with DODAB LUVs presents Soret band peaking at 403 nm with a shoulder at 392 nm. In the presence of DODAB LUVs, the Q-band region exhibits a peak at 570 nm.

### 2.3. Magnetic Circular Dichroism Spectral Features of Cu(II) and Fe(III) MP-11 in Homogeneous and Heterogeneous Media

**Figure 4:**
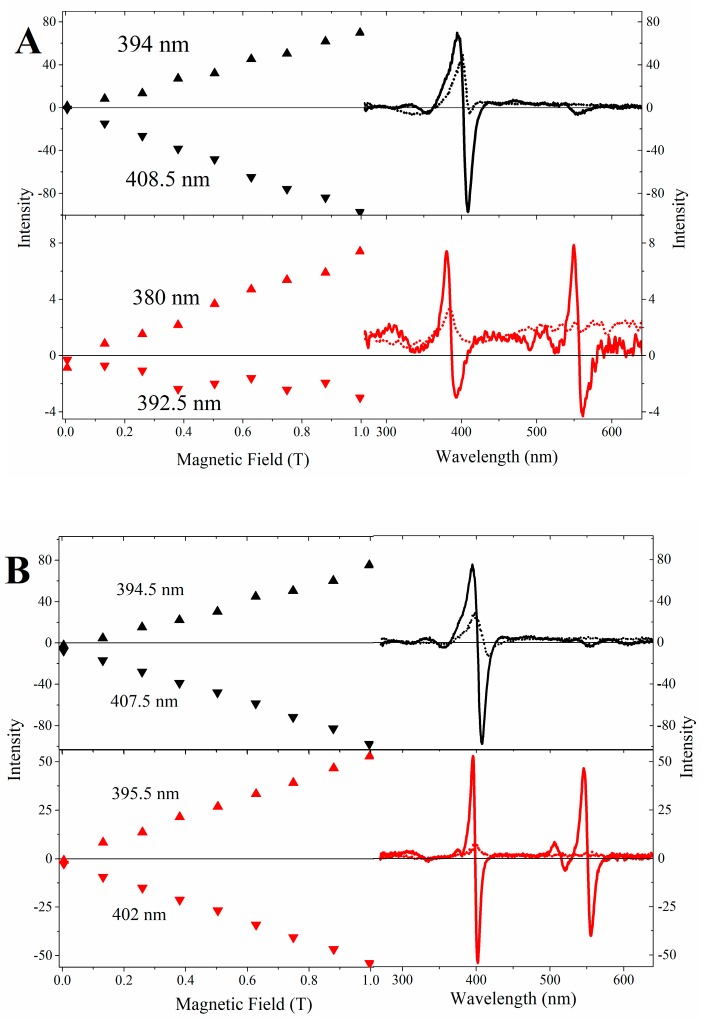

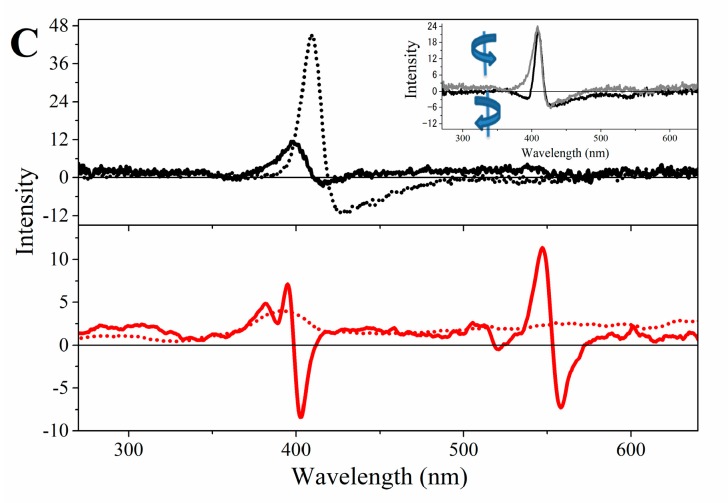
Comparative CD and MCD spectra of Fe(III) and Cu(II)MP-11 in homogeneous and heterogeneous media. (**A**) HEPES buffer. Left panel corresponds to positive and negative λmax at increased values of the external magnetic field (from 0.005 to 0.9965 T). Black and red symbols correspond to (Fe(III) and Cu(II), respectively. Right panels, depicted the CD spectra (dotted lines) of Fe(III) (black lines) and Cu(II)MP-11 (red lines) superimposed by the corresponding MCD spectra (solid lines); (**B**) CTAB micelles. Left panel corresponds to positive and negative λmax at increased values of the external magnetic field (from 0.005 to 0.9965 T). Black and red symbols correspond to (Fe(III) and Cu(II), respectively. Right panels, depict the CD spectra (dotted lines) of Fe(III) (black lines) and Cu(II)MP-11 (red lines) superimposed by the corresponding MCD spectra (solid lines) and (**C**) DODAB LUVs. Upper and lower panels show the CD spectra (dotted lines) of Fe(III) (black lines) and Cu(II)MP-11 (red lines) superimposed by the corresponding MCD spectra (solid lines). In the upper panel of C, the inset shows a non-typical equally intense band obtained at positive (gray line) and negative (black line) magnetic fields. The spectra were recorded with 20 µM MP-11 using a 0.1 cm quartz cuvette at 25 °C.

Fe(III) and Cu(II)MP-11 in HEPES buffer and associated with positively charged interfaces were also analyzed by CD and MCD techniques (Figure 4A–C). The spectra shown in Figure 4A were run under increasing values of the external magnetic field produced by an electromagnet set with increasing electric current values in the range of 0.005 to 0.9965 T. In each fixed magnetic field, the spectra were run at positive and negative magnetic field direction modes. The subtraction of the spectra obtained in the positive mode of the magnetic field from the corresponding spectra obtained in the negative mode resulted in pure MCD spectra that are represented by red lines. The sum of the corresponding spectra obtained at the positive and negative modes of each magnetic field gives pure CD spectra that are represented as black lines. Figure 4A shows, respectively, in the right, upper and lower, panels, the CD spectra of Fe(III) and Cu(II)MP-11 in HEPES buffer (dotted black and red lines, respectively). The CD spectra represented as dotted lines are superimposed by the corresponding MCD spectra (solid lines).

Metal porphyrins in cytochrome c and microperoxidases, exhibit CD and MCD signals. In these complexes, the presence of a set of *d* orbitals that are partially filled permit ligand to metal and metal to ligand transitions responding for the charge transfer bands (LMCT and MLCT) that are present in addition to the π→π* bands. The presence of CD signals results from the zero-field splitting that is a complete lifting of *d* orbital degeneracy promoted by a heme iron rhombic distortion (*x* ≠ *y* ≠ *z*) and modulated by the strength of the axial ligands.

The Fe(III)MP-11 CD spectra (Figure 4A, black line) exhibits the Soret band with an absolute predominance of the positive signal. The absence of a corresponding negative signal is consistent with a weak field ligand at the sixth coordination position of the heme iron or a pentacoordinate species [16,31]. The Soret band of Fe(III)MP-11 is split under the external magnetic field applied and exhibits positive and negative signals. The MCD spectrum has the zero crossing at 401.5 nm that slightly diverged of the Soret band peak shown by the corresponding electronic absorption spectrum (peak at 400 nm). The MCD positive and negative bands of Fe(III)MP-11 are not featured as specular image each other. These spectral features are consistent with the contribution of a Faraday A-term overlapped by a B-term contribution. The Faraday A-term contributes with a derivative MCD signal that has the zero-crossing coincident with the peak of the Soret band of the electronic absorption spectrum. The overlapped contribution of a Faraday B-term affects the intensity and bandwidth of the positive and negative components of the derivative signal and mismatch the zero crossing from the Soret band peak. The MCD intensity increased linearly with the magnetic field strength (Figure 4A, left panel). The Cu(II)MP-11 CD spectrum (Figure 4A, dotted red line) presents a relatively low intensity in comparison with the Fe(III)MP-11 species (Figure 4A, dotted black line). In the Cu(II)MP-11 CD spectrum, the Q bands are not detectable, but the spectrum intensity was proportionally increased according to the strength of an external magnetic field applied (Figure 4A, lower panels). The association of Fe(III) and Cu(II)MP-11 with CTAB micelles (Figure 4B) promoted a significant increase in the MCD spectra intensity as compared with the spectra obtained in HEPES buffer. The association of Fe(III)MP-11 with DODAB vesicles favored the intensity of the CD signal significantly. Figure 4C shows that differently of the results obtained in HEPES and CTAB micelles, in DODAB vesicles the CD signal is considerably more intense than the MCD one. The inset of Figure 4C, upper panel, shows non-typical equally intense bands obtained at positive (gray line) and negative (black line) magnetic fields. These atypical features are promoted by the high contribution of the CD signal that becomes evident by subtracting the signals obtained at positive and negative modes of the magnetic fields. Cu(II)MP-11 associated with DODAB vesicles presented a broad CD Soret band and weak spectral features in the Q-band region (Figure 4C, black line). The MCD spectrum of Cu(II)MP-11 exhibits a complex Soret band featured by the overlapped contribution of Faraday A and B terms. In the visible region, Cu(II)MP-11 associated with DODAB vesicles presented a relatively strong asymmetrical sigmoidal band. The region between the Soret and Q bands showed weak spectral features that are assigned to metal-ligand charge transfer transitions.

### 2.4. EPR Spectral Characteristics of Cu(II) and Fe(III) MP-11 in Homogeneous and Heterogeneous Media

**Figure 5:**
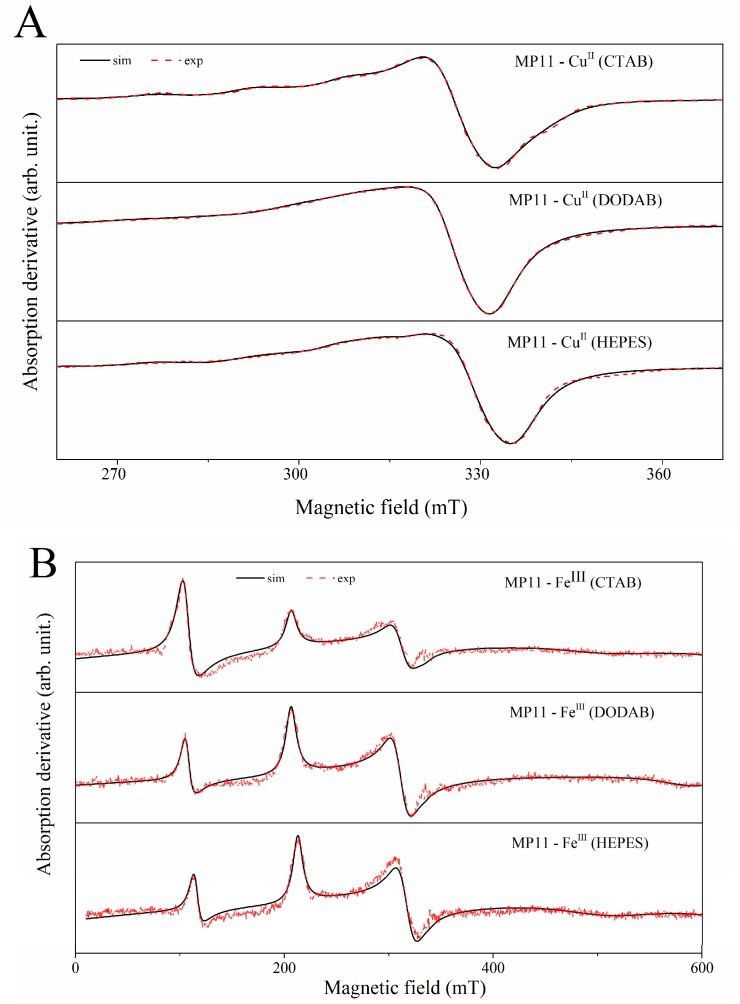
Experimental and simulated EPR spectra of MP-11 with Fe(III) (**A**) and Cu(II) (**B**) as the central metals in HEPES, CTAB, and DODAB, as indicated and obtained with frozen solutions of same concentrations. The EPR parameters used are shown in Table 2.

The interaction of Fe(III)MP-11 and Cu(II)MP-11 with the positively charged interfaces of CTAB micelles and DODAB vesicles were also analyzed via direct CW-EPR measurements and simulations (Figure 5).

The parameters used to simulate the experimental EPR spectra are shown in Table 2.

Two distinct paramagnetic species were determined for both Fe(III) and Cu(II)MP-11 in homogeneous and heterogeneous media. Cu(II)MP-11 is an S = ½ species in HEPES buffered water as well as when it is associated with CTAB micelles and DODAB vesicles (Figure 5A,B). The EPR spectra of Fe(III)MP-11 in homogeneous and heterogeneous media presented the contribution of high (S = 5/2) and low spin (S = 1/2) species. However, in HEPES-buffered water and associated to lipid nanostructures, the low spin form was predominant for Fe(III)MP-11. The predominance of Fe(III)MP-11 low spin form is evident because the EPR absorption of heme iron in the high spin state is significantly higher when compared with the same concentration of heme protein in the low spin form. The presence of both low and high spin Fe(III)MP-11 indicates different ligands at the sixth coordination position of heme iron. Strong field ligand causes a higher energy splitting between *t_2g_* and, e.g., states of *d* orbitals in cubic symmetries [32] leading to the particular population of *t_2g_* states. For MP-11, strong field ligands are provided by intra- and inter-chain coordination. Conditions that promotes disaggregation of MPs favors the increase of the high spin form and the low spin form can occur only by intra-chain coordination. The mathematical areas under the high and low spin EPR signals were calculated, and the low spin area/high spin area ratios were: 6.5 for HEPES buffer, 5.4 for DODAB vesicles and 2.8 for CTAB micelles. Therefore, the association with CTAB micelles was the condition that more efficiently favored the disaggregation of Fe(III)MP-11. The monomeric form has previously been reported to have a high spin configuration in immobilized MP-11 [33]. The EPR spectra of Cu(II)MP-11 were useful also to demonstrate the heme peptide disaggregation in micelles, although for a different reason: the spectrum of the sample has a better-resolved Cu(II) (I = 3/2) hyperfine structure in CTAB micelles than in HEPES-buffered water and in DODAB vesicles. The broadening of the EPR signal as a consequence of dipolar interaction is consistent with the proximity (aggregation) between the metallic centers as pointed out by the other experimental techniques used in this study. The highly anisotropic Fe(III) low spin species in MP-11 is consistent with a distortion of the heme group. The strength of the rhombic (*V*) versus the axial field (*∆*), *V/∆*, is 0 for the perfectly axial system and ~0.67 for a simple rhombic system. They can be calculated from the g-tensor values according to Zopperallo et al. [32]. For Fe(III)MP-11, *V/∆* was calculated to be 0.46 in this study, which corresponds to a distorted axial system.

**Figure 6:**
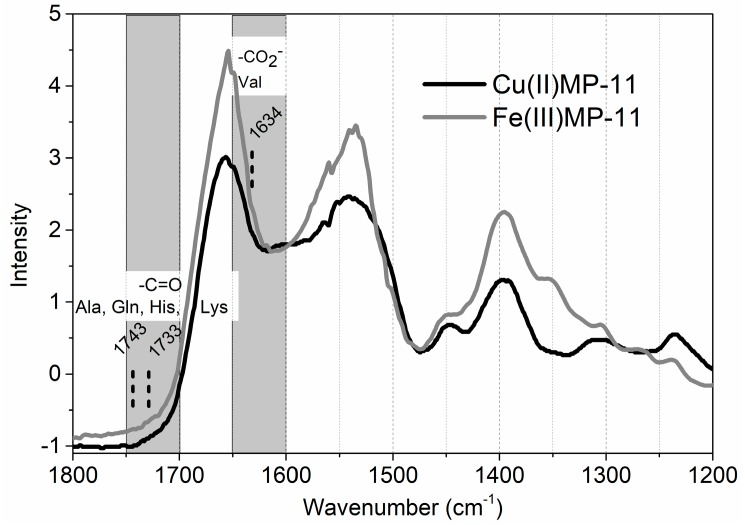
FTIR spectra of Cu(II) (black line) and Fe(III)MP-11 (gray line) and the spectral regions where the vibrational bands of Cu(II) complexed with carbonyl groups of Ala, Gln, His and Lys and the region of Val complexed with Cu(II) are expected.

The spectral characterization of Cu(II)MP-11 was consistent with Cu(II)-heme complex rather than Cu(II)-peptide chain due to the following characteristics: (i) the absence of a broad absorbance band at the spectral region of 500–800 nm overlapping the Q bands [34]. The high intensity and sharp Soret band at the spectral region of 400 nm resulting from an allowed electronic transition as well the low intensity Q bands around 500 nm are typical of metal porphyrin spectra [35]. Particularly the absorbance and MCD spectra of Cu(II)MP-11 in CTAB micelles show clearly the exclusive contribution of Cu(II)-porphyrin in the visible region; (ii) the absence of Cu(II)-peptide complexes was also reinforced by EPR spectrum that was consistent with Cu(II)-porphyrin and not with Cu(II)-peptides. It is important to note that the acidic pH condition in which the metalation of free base MP-11 was done, is not favorable for the formation of Cu(II)-peptide complexes. Despite the strong pieces of evidence of the absence of Cu-peptide complexes, FTIR analysis of Cu(II)MP-11 in comparison with Fe(III)MP-11 was also performed (Figure 6).

Figure 6 shows the FTIR spectra of Cu(II) (black line) and Fe(III)MP-11 (gray line) and the spectral regions where the vibrational bands of Cu(II) complexed with carbonyl groups of Ala, Gln, His and Lys and the region of Val complexed with Cu(II) are expected [34]. However, the α-carboxylic group of Val is not available for coordination with Cu(II) since it is involved in a peptide ligation. The spectra of Cu(II) and Fe(III)MP-11 are very similar e no bands are present in the spectral region of Cu-amino acid complexes, considering the amino acid residues of MP-11 chain.

### 2.5. Catalytic Properties of Cu(II) and Fe(III)MP-11 Homogeneous and Heterogeneous Media Using Amplex^®^ Red

The catalytic properties of Fe(III) and Cu(II)MP-11 challenged by peroxides were investigated by UV-visible spectral changes of Soret and Q bands and using Amplex^®^ Red. One-electron oxidation of the colorless compound Amplex^®^ Red produces the pink and fluorescent dye resofurin by the mechanism of radical disproportionation [36].

The investigation of the reaction of Fe(III)MPs with hydrogen peroxide and *t*-BuOOH by electronic absorbance spectroscopy and EPR has shown the formation of Compound II (Fe(IV)=O) as the high valence intermediate species of the catalyst. In these reactions, the generation of excited species and free radicals promote progressive bleaching of MPs [37]. The effect of CTAB on the reaction of Fe(III)MP-9 with hydrogen peroxide and *t*-BuOOH was also previously investigated. In the presence of CTAB, the catalytic efficiency of the reaction was limited by the hydrophobicity of the substrate, i.e., the reaction of organic peroxides is facilitated in CTAB by the preferential partition of these substrates in the micelle core. Here, the study of the peroxidase activity of Fe(III)MP-11 in buffered water, CTAB and DODAB was extended for the peroxidase cycle using Amplex Red^®^ as the reducing agent. For comparison, corresponding experiments were carried out with HRP (Figure 6 and Table 1). MP-11 and HRP kinetic parameters in buffered water, CTAB and DODAB were determined by treating the data by Equations (1) and (2). In the Michaelis-Menten model, the initial rate of the enzymatic reaction (*V*) is related to the concentration of substrate [*S*], the rate attained at conditions of enzyme saturation, *V_max_* and the Michaelis constant, *K_m_*. The kinetic curves of absorbance increasing at 571 nm (resofurin production) was measured after the addition of hydrogen peroxide (2–20 µM) to a solution of 25 nM HRP or 100 nM Fe(III)MP-11 and 40 µM Amplex Red^®^, in buffered water, CTAB micelles, and DODAB vesicles. The rate constant (*k_obs_*) was determined for each hydrogen peroxide concentration (2–20 µM) by adjusting the curves of resofurin production to Equation (1):

(1) $$y=a\left(1-e^{-k_{obs}t}\right).$$

The increase of absorbance at 571 nm obeys an exponential relationship that allows the fit to the first order equation. In Equation (1), *y* and *a* are, respectively, the absorption intensity of resofurin at a determined time (*t*) and at the infinite time and *k_obs_* is the individual observed rate constant for resofurin production. Figure 6 shows representative kinetic curves of resofurin production obtained with Fe(III)MP-11 associated to CTAB micelles, using 2, 5 and 10 µM of hydrogen peroxide.

The Lineweaver-Burk plot is obtained by fitting the data according to Equation (2) that yields *K_m_* and *V_max_*:

(2) $$\frac{1}{k_{obs}}=\frac{K_{m}}{V_{max}}\times \frac{1}{\left[S\right]}+\frac{1}{V_{max}}.$$

**Figure 7:**
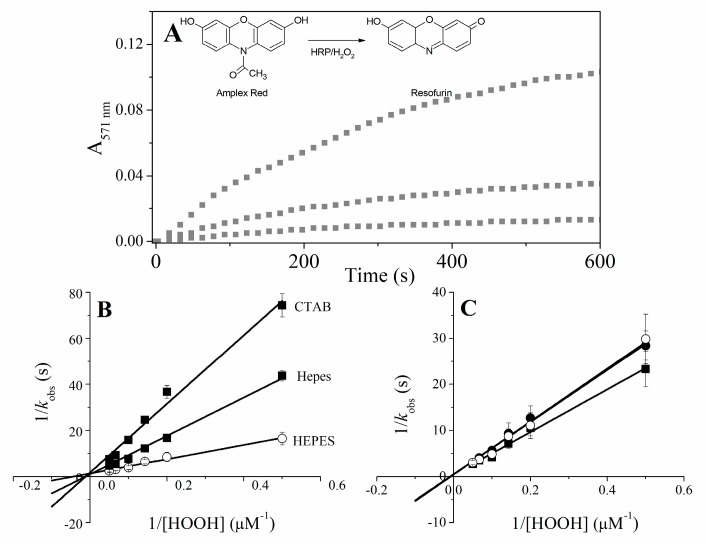
Kinetic study of the reaction of Fe(III)MP-11 and HRP with Amplex Red^®^ as a function of hydrogen peroxide concentration. (**A**) Representative curves of resofurin production during the Amplex Red^®^ oxidation by Fe(III)MP-11 associated with CTAB micelles, using 2, 5 and 10 µM of hydrogen peroxide; (**B**) Fe(III)MP-11 and (**C**) HRP Lineweaver-Burk plots of the reciprocal *k_obs_* values obtained by fitting the curves of temporal generation of resofurin vs. the reciprocal of the hydrogen peroxide concentration. The inset of panel A shows the structures of Amplex Red^®^ and resofurin. Experimental conditions were: 100 nM Fe(III)MP-11 or 25 nM HRP, hydrogen peroxide concentrations were used in the range of 2–20 µM and 40 µM Amplex Red^®^ buffered by HEPES. The reactions were carried out in a homogeneous medium (solid squares), 20 mM CTAB (solid circles) and 2 mM DODAB (open circles).

**Figure 8:**
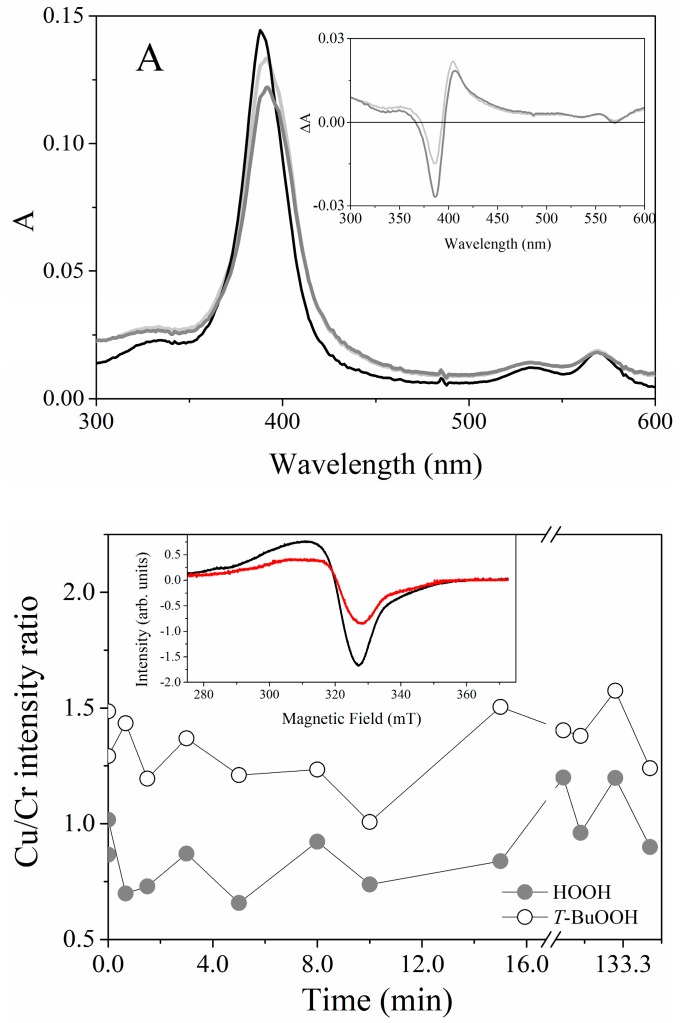
Cu(II)MP-11 peroxidase activity. (**A**) Time-resolved UV-visible spectra of 1 µM Cu(II)MP-11 during the reaction with 0.8 mM HOOH at 5 mM HEPES buffer solution, pH 7.4. The decreasing intensity spectra correspond to zero, 12 (light gray line) and 15 min (gray line) after hydrogen peroxide addition. The inset corresponds to the differential spectra obtained by subtracting the initial time from the spectra obtained after 12 min (light gray line) and 15 min (gray line) of the reaction; (**B**) EPR from Cu(II)MP-11 peroxidase activity using Cr(III) as a signal marker. The reactions were done in the presence of HOOH (gray circle) or *t*-BuOOH (open circle). The inset shows the first spectra (time zero) and after 70 min of *t*-BuOOH addition. Experimental conditions: 0.1 mM Cu(II)MP-11, 0.3 mM of peroxides, water buffered solution, gain 50 dB, modulation amplitude 1 mT, power microwave 2.5 mW, frequency microwave 9.48 GHz, the time constant 20 ms, conversion time 81.92 s, temperature 10 K.

The *k_cat_* values are obtained by the division of the *V_max_* by the enzyme concentration (Table 3). Table 3 shows that the association with positively charged interfaces decreased around 20% the *k_cat_* of HRP. For Fe(III)MP-11, CTAB increased around 25% the *k_cat_* and DODAB had the opposite effect, i.e., decreased the *k_cat_* around 25%. DODAB and CTAB tripled the *K_mapp_* of Fe(III)MP-11, the effect of DODAB > CTAB. In the case of HRP, the effects of DODAB and CTAB on the affinity for the substrate were similar but discrete. Consistently, the positively charged lipids decreased the catalytic efficiency of Fe(III)MP-11 and HRP, but the effects were more pronounced for the former catalyst. The catalytic behavior of Cu(II)MP-11 was peculiar. The reaction of Cu(II)MP-11 with hydrogen peroxide was analyzed by electronic absorption spectroscopy (Figure 7), the Amplex Amplex Red^®^ assay (Figure 7B) and EPR (Figure 8). Figure 7A shows the spectra of Cu(II)MP-11 before (black line), 12 min (light gray) and 15 min (gray line) after the addition of hydrogen peroxide. The inset shows the differential spectra obtained by subtracting the initial time from the spectra obtained after 12 min (light gray) and 15 min (gray line) of the reaction.

The spectral changes of Cu(II)MP-11 observed in the course of the reaction with hydrogen peroxide (Figure 8A) and *t*-BuOOH (not shown) demonstrated the catalytic activity of the heme peptide against peroxides. EPR experiments demonstrated the cyclic nature of the reactions of Cu(II)MP-11with both hydrogen peroxide and *t*-BuOOH. Figure 8B shows the cyclic variation of the signal intensity of Cu(II) determined via the Cu(II)/Cr(III) signal ratio during the reaction of Cu(II)MP-11with both hydrogen peroxide (gray symbol) and *t*-BuOOH (white symbol).

The signal of Cr(III), as a standard intensity signal, was measured concomitantly in an encapsulated capillary into a quartz tube together with the sample. Considering that Cu(I) is EPR silent, the oscillation in the Cu(II) signal intensity is consistent with a mechanism similar to that proposed for SOD-1 peroxidase activity. Bonini et al. demonstrated that SOD-Cu(II)/Zn can oxidize hydrogen peroxide to superoxide ion [38]. The SOD-Cu(I) recycles to the Cu(II) form using peroxycarbonate as the reducing agent. In aqueous medium, peroxycarbonate is formed from the reaction of hydrogen peroxide with carbon dioxide. Alternatively, superoxide ion can recycle SOD-Cu(I) to the resting form (Cu(II)). The reactions contributing for a potential cyclic behavior Cu(II)MP-11 reacting with peroxides are depicted in the following Equations (3)–(7):

Cu(II)MP-11 + HOOH → Cu(I) − MP-11 + O_2_^•^^−^ (3)

Cu(II)MP-11 + O_2_^•−^ → Cu(I) − MP-11 + O_2_ (4)

HOOH + CO_2_ → HOOCO_2_ (5)

Cu(I) − MP-11 + HOOH → Cu(II)MP-11 + OH^•^ + H_2_O (6)

Cu(I) − MP-11 + HOOCO_2_ → Cu(II)MP-11 + ^•−^CO_3_ + H_2_O (7)

**Figure 9:**
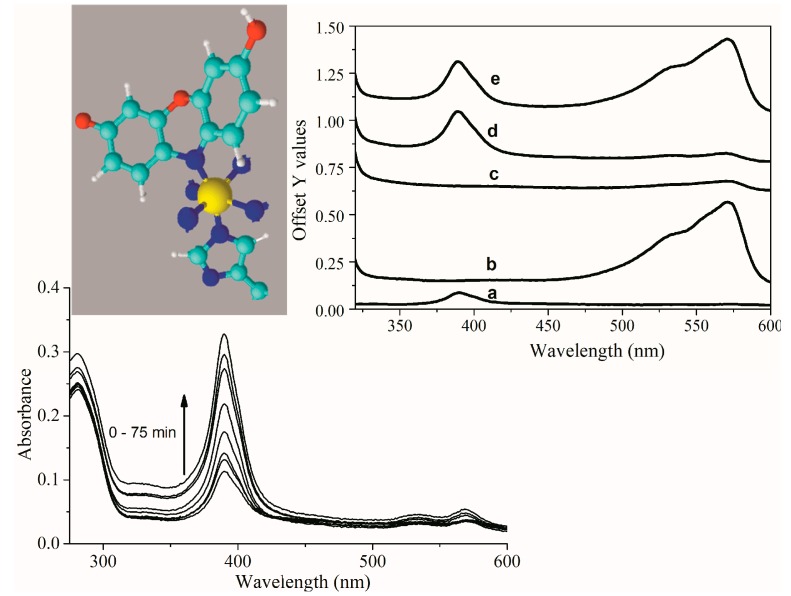
Reaction of the hydrogen peroxide-activated Cu(II)MP-11 with Amplex Red. Time scan in the time range of 0–75 min of 0.5 µM Cu(II)MP-11 after addition of 40 µM Amplex Red and hydrogen peroxide, as indicated by the arrow. The inset shows the spectra of: (**a**) 0.5 µM Cu(II)MP-11; (**b**) Resofurin produced by 25 nM HRP activated by 40 µM hydrogen peroxide; (**c**) Leucoresofurin produced by resofurin reduction by superoxide ion generated by xanthine/xanthine oxidase system in the presence of 25 nM catalase used to eliminate hydrogen peroxide produced by spontaneous ion superoxide dismutation; (**d**) Cu(II)MP-11 added to leucoresofurin solution; (**e**) the same conditions of (**d**) reoxygenation after agitation. Another inset shows the structural representation of dihydroresofurin in a putative coordination with Cu(II) in the porphyrin ring. Only nitrogen atoms of the porphyrin ring were represented for clarity. In the 3D structure, carbons are cyan, nitrogens are blue, copper is yellow and oxygen is red. MP-11 histidine residue was represented at the fifth coordination position of cupper ion. Xanthine concentration was 400 µM and xanthine oxidase 0.6 U/mL.

A similar mechanism could be operated by Cu(II)MP-11. In an air-equilibrated system, cyclic reactions of Cu(II)MP-11 with hydrogen peroxide could be observed. The catalytic activity of Cu(II)MP-11 was also investigated by the Amplex Red^®^ assay as shown in Figure 9, which shows the spectral changes of Cu(II)MP-11 observed after addition of hydrogen peroxide and Amplex Red.

Differently from Fe(III)MP-11, the spectrum of resofurin with peak at 571 nm did not appear during the incubation of the copper heme peptide with hydrogen peroxide and Amplex Red. In addition, a time-dependent increase of Cu(II)MP-11 Soret and Q bands during the reaction with Amplex Red was observed. Nims et al. [39] reported the formation of a leucoresofurin, i.e., dihydroresofurin, by cytosolic reducing agents. More recently, Song et al. [40] calculated the structure of dihydroresofurin produced by using NaBH_4_ as resofurin reducing agent. The progressive increase of MP-11 absorbance intensity without the appearance of resofurin visible band suggested that free radicals produced by hydrogen peroxide cleavage could oxidize Amplex Red to resofurin. In sequence, superoxide ion produced in Equation (3) could reduce resofurin to dihydroresofurin that does not absorb in the visible range. The abovementioned mechanism implicates that superoxide ion can reduce resofurin and that dihydroresofurin is able to increase the Soret and Q bands of Cu(II)MP-11, probably by coordination of its nitrogen atom with Cu(II). To investigate the proposed mechanism, resofurin was previously generated by HRP activated by hydrogen peroxide (inset of Figure 9, line b) and challenged by superoxide ion produced by xanthine/xanthine oxidase system [41] (inset of Figure 9, line c). Exposure to superoxide ion promoted bleaching of resofurin. The bleaching promoted by superoxide ion was reversed by re-oxygenation of the medium (not shown). Cu(II)MP-11, in the concentration of 0.5 µM, was added to dihydroresofurin produced by superoxide ion and, in this condition, exhibited a significant increase of absorbance when compared with its spectrum determined in HEPES buffer (inset of Figure 9, line a). After addition of Cu(II)MP-11, the agitation of the solution led to the appearance of resofurin band but without recover spectral features of the copper enzyme spectrum to that exhibited in line a of Figure 9. The concentration of Cu(II)MP-11 corresponds to ~1% of the initial Amplex Red^®^. concentration, therefore even 100% of the enzyme coordinated dihydroresofurin, the concentration of the free form of reduced resofurin should not be significantly affected. The recovering of resofurin color without changes in the spectrum of Cu(II)MP-11 suggests that dihydroresofurin is stabilized in the reduced form when coordinated with the enzyme. Another possibility that may not be discarded is a chemical modification of Cu(II)MP-11 by a secondary reaction with dihydroresofurin leading to the observed spectral changes. The complete elucidation of the reaction mechanism of Cu(II)MP-11 with Amplex Red deserves future investigations.

## 3. Discussion

**Figure 10:**
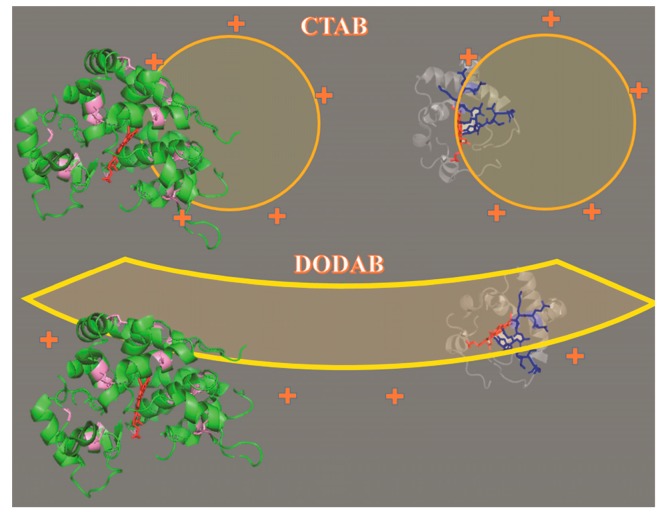
Cartoon of putative interactions of HRP and MP-11 with CTAB micelles and DODAB vesicles. HRP and cytochrome c structures were generated in PyMol 4.3software, using the respective data for 1H58 and 1HRC that are available in Protein Data Bank. The MP-11 structure was highlighted in blue in the structure of cytochrome c (transparent gray). The structure of HRP (green) has the acidic amino acid residues that are responsible for the affinity to the positively charged interfaces highlighted in light cyan.

Numerous applications of microperoxidases have been described in the literature. The versatility of the microperoxidases as catalysts is provided by the coordination sphere of the heme iron, by different solvents, by the association with lipid membranes, mesoporous silica, and by the presence of exogenous axial ligands. More recently, an activity of technological importance, the production of hydrogen, was described for microperoxidase that had the iron ion, its central metal, replaced by cobalt ion. Thus, the present study aimed to produce and characterize copper microperoxidase and to analyze how the association with positively charged lipid interfaces could interfere in its structure. The tryptic excision of a large extension of cytochrome c polypeptide chain converts this heme peptide, a basic protein, into an acid peptide, which is negatively charged at pH values above 5. This characteristic endows microperoxidases with affinity for positively charged interfaces that here was provided by the CTAB and DODAB surfactants, organized respectively as micelles and LUVs. The analysis of colloidal stability of CTAB micelles and DODAB LUVs associated with Cu(II)MP-11 and Fe(III)MP-11 at low and high temperatures showed that the metal MPs establish electrostatic and hydrophobic interactions with surfactants. The hydrophobic interaction of Cu(II)MP-11 with DODAB is dependent of the bilayer fluidity since the colloidal stability was achieved above the phase transition temperature. DODAB vesicles are stable and do not exchange surfactant monomers with each other. Therefore, MP molecules electrostatically attached to the vesicle surface might insert themselves through the bilayers under high fluidity conditions. On the other hand, MP was added to previously organized CTAB micelles that are in equilibrium with monomers. The increase in temperature shifts the equilibrium towards the monomers that can reorganize a supramolecular structure around MP leading to colloidal stability. The interaction of Cu(II) and Fe(III)MP-11 with positively charged interfaces modulate their peroxidase activity. The kinetic results obtained for Fe(III)-MP associated to DODAB vesicles, and CTAB micelles demonstrated which, in these conditions, several factors influence the peroxidase activity of metal MPs. Spectroscopic analysis of the metal MPs associated to DODAB and CTAB showed the occurrence of structural changes that have repercussion on the enzymatic activity. However, the comparison of the kinetic data obtained for Fe(III)-MP and HRP demonstrated that the topology of the peptide in the micelles and liposomes also influences the enzymatic activity. HRP bears a significantly higher structure than the MP with heme group buried in the apoprotein. The dimensions and hydrophilicity of HRP impair the enzyme insertion in DODAB bilayers. Therefore, HRP should remain on the surface of CTAB micelles and DODAB vesicles leading to a similar effect of both the surfactants on its activity. Fe(III)MP-11, a low molecular weight peptide with the heme group exposed to the environment and with a more hydrophobic structure could interact differently with CTAB micelles and DODAB vesicles. The interaction with DODAB vesicles only achieved after heating probably results in the heme group buried inside the bilayers and poorly accessible to the substrates. The interaction of Fe(III)MP-11 with CTAB micelles probably results in more easy accessibility to the substrates than when associated with DODAB vesicles. The peculiar interactions of MP-11 with CTAB micelles and DODAB liposomes respond for the corresponding decrease of catalytic efficiency observed in the presence of these surfactants. Figure 10 shows a cartoon illustrating the structural differences between HRP and MP-11 leading to different interactions with the DODAB vesicles and CTAB micelles. The presence of Cu(II) as the central metal of MP-11 changes the catalytic behavior of the heme peptide and a probable SOD mimetic activity was observed. However, the present results demonstrated that the catalytic activity of microperoxidases could be finely modulated by the microenvironment and the type of the central metal. A systematic investigation of the catalytic mechanism of Cu(II)MP-11 on peroxides, Amplex Red^®^ and other potential substrates are the future research directions for the present study.

## 4. Materials and Methods

### 4.1. Chemicals

MP-11, CTAB, DODAB, and copper sulfate were from Sigma Chemical Co. (St. Louis, MO, USA). All aqueous solutions were prepared with Milli-Q water quality. The pH was measured using a combined glass electrode (Orion Glass pH SURE-FLOW, Thermo Fisher Scientific Inc., Waltham, MA, USA). The buffer solutions were prepared with 5 mM of 4-(2-hydroxyethyl)-1-piperazineethanesulfonic acid (HEPES) adjusted to pH 7.4.

### 4.2. Preparation of Cu(II)MP-11 [42]

The procedure for the preparation of metal-free porphyrin with anhydrous HF has been described previously [43]. The method developed for copper insertion is accomplished simply through dialysis at 4 °C of a freshly prepared MP-11 free metal solution (0.2 mM) against a 1.25 mM solution of cupric sulfate (Sigma). The reaction is complete in 4 h, as evidenced by the disappearance of the four characteristic absorption bands of metal-free porphyrin in the visible wavelength region [3,5]. Excess cupric sulfate is removed by dialysis against water overnight, with at least three changes of distilled water.

### 4.3. Preparation of the CTAB Micelles and Large DODAB Vesicles

CTAB was purified as previously described [44] and then weighted to prepare 20 mM and 60 mM stock dispersions in 5 mM HEPES buffer by stirring at 37 °C. Large unilamellar vesicles (LUVs) of DODAB were obtained from the DODAB powder without further purification. Large vesicles have been achieved as previously described with 2 mM DODAB in 5 mM HEPES by heating and vortexing by 10 min above the temperature of DODAB bilayer gel-to-liquid-crystalline phase transition (70 °C) [45,46]. This procedure yields LUVs with a mean diameter around 400 nm [47]. For samples in which MP-11 was added, the LUVs were incubated with the hemepeptide at 70 °C (for bilayers in the liquid-crystalline state) for 30 min to facilitate the peptide incorporation inside the LUV bilayer.

### 4.4. Zeta-Potential Measurements

The zeta-potential of the complexes were measured by a Nano-ZS Zetasizer (Malvern, Worcestershire, UK). Zeta potential measurements were performed using a folded capillary cell (DTS1060) made of polycarbonate with gold plated beryllium/copper electrodes. The measurements were obtained with samples used for magnetic circular dichroism measurements (MP-11/CTAB micelles and MP-11/large DODAB vesicles complexes) after fivefold dilution with the respective buffer.

### 4.5. Determination of UV-Visible Optical Spectra

Absorption spectra were recorded using an Evolution™ Array UV-Visible Spectrophotometer (Thermo Fisher Scientific Inc., Waltham, MA, USA) in the photodiode array scan mode. The spectral resolution was 0.5 nm. The optical path length was 0.1 cm at room temperature.

### 4.6. Magnetic Circular Dichroism (MCD)

MCD spectra (300–650 nm) were obtained at room temperature using a J-815 spectropolarimeter (Jasco, Tokyo, Japan). All MCD spectra were subtracted from their respective baselines. The optical path length was 0.1 cm, the MP-11 concentration was around 20 µM, and the magnetic field varied from 0.005 to 0.995 T.

### 4.7. Determination of Heme Fe(III) and Cu(II) EPR Spectra

EPR spectra of MP-11 native and metal substituted (100 µM) were recorded using an X-band spectrometer (Elexsys line E-580, Bruker, Billerica, MA, USA) equipped with a standard rectangular cavity. The temperature of 4 K was maintained by a low-temperature accessory (Helitran, Oxford Systems, Abingdon, Oxfordshire, UK). The sample was placed in a quartz tube and frozen in liquid nitrogen before being introduced into the microwave cavity. Spectra for blank samples (buffer only) were subtracted from MP-11 containing samples, taking into account the matching of microwave frequency. Spectral parameters were fixed as follows: the microwave frequency at 9.5 GHz, the microwave power at 8 mW, the magnetic field scan range from 50 up to 350 mT, and the modulation amplitude at 1 mT. EPR spectra were simulated using the Easyspin program, a MATLAB toolbox, widely used to simulate EPR powder spectra and both Gaussian and Lorentzian line shapes.

### 4.8. FTIR Spectroscopy

Infrared spectra were recorded using a 640-IR spectrometer (Varian, Santa Clara, CA, USA) fitted with a DLaTGS detector and a MIRacle™ single reflection horizontal ATR accessory (PIKE, Madison, WI, USA,) with around 1.8 mm of sampling área diamond/ZnSe crystal and 45 degrees angle of incidence. A series of cumulative spectra were measured corresponding after 15 uL of the sample had been dropped in contact with the crystal and let drying at room temperature (20 ± 1 °C for 30–40 min. These thin films as shaped have sufficient optically sampled surface area to enable good quality infrared spectra to be obtained from a single internal reflection. The reference spectrum was the crystal. All spectra were calculated from 64 scans at 4 cm^−1^ resolution and were not corrected for the wavelength dependence of absorbances.

### 4.9. Amplex^®^ Red Oxidation

HRP and Amplex^®^ Red were purchased from Sigma. The concentration of HRP was determined spectrophotometrically at 403 nm (ε = 1.02 × 105 M^−1^ cm^−1^) [42,48]. Relationship between the yield of oxidation of Amplex^®^ Red by H_2_O_2_ in the presence of the HRP, Cu(II)MP-11 and Fe(III)MP-11, was determined by measuring absorbance intensity due of resorufin formed. Under all conditions, 5 mM pH 7.4 HEPES, 20 mM CTAB and 2 mM DODAB vesicles, containing 40 µM Amplex Red, 25 nM HRP or 100 nM Fe(III)MP-11, 20 µL of hydrogen peroxide solution (2–20 µM) was added with a multichannel micropipette. Absorbances at 571 nm were measured after an automatic brief shaking at 30 °C in a microplate reader (Synergy HT, BioTek Instruments, Winooski, VT, USA). The reactions were done in a total volume of 200 μL per microplate well, in triplicate for each H_2_O_2_ concentration. The concentration of resorufin formed was calculated assuming the molar absorption coefficient at 571 nm of 6.3 × 10^4^ M^−1^ cm^−1^ [43,49].

## 5. Conclusions

The acidic peptide MP-11 produced by the tryptic digestion of cytochrome c has binding affinity to positively charged interfaces provided by CTAB micelles and DODAB liposomes. The primary electrostatic binding of MP-11 with CTAB and DODAB is followed by different degrees of peptide insertion inside the hydrophobic regions of the micelles and bilayers. The differentiated interaction of Fe(III)MP-11 with CTAB micelles, and DODAB vesicles result in structural changes and a decrease of the heme group accessibility to substrates. Cu(II)MP-11 presented a catalytic behavior that suggests a Cu/Zn-SOD mimetic catalyst and that will be the focus of future investigations.

## Figures and Tables

**Table 1:** *Zeta*-potential (ξ) of DODAB LUVs and CTAB micelles in the presence and the absence of Fe(III) or Cu(II)MP-11.

MP-11	ξ (mV)
Cu(II) MP-11	−27.8 ± 3
Fe(III) MP-11	−13.1 ± 5
CTAB micelles	+50.5 ± 2
Cu(II)MP-11/CTAB micelles	+34.3 ± 1
Fe(III)MP-11/CTAB micelles	+46.1 ± 3
DODAB vesicles	+65.7 ± 6
Cu(II)MP-11/DODAB vesicles	+61.6 ± 7
Fe(III)MP-11/DODAB vesicles	+57.1 ± 4

**Table 2:** EPR parameters obtained from simulation of experimental spectra using Easyspin.

			Cu(II)MP-11							Fe(III)MP-11				
	Medium	g_x_	g_y_	g_z_	A_x_ (MHz)	A_y_ (MHz)	A_z_ (MHz)	ΔH (mT)	%	g_x_	g_y_	g_z_	ΔH (mT)	%
Sp.1	HEPES	2.04	2.11	2.25	60	108	482	8.5	44	5.89		2	12.5	0.09
	CTAB	2.05	2.08	2.24	38	56	466	2	63	5.95		2	18.2	0.54
	DODAB	2.04	2.09	2.31	54	70	356	17	61	5.88		2	12.7	0.09
Sp.2	HEPES	2.04	2.06	2.19	11	53	237	11	56	1.32	2.13	3.18	10.8	99.91
	CTAB	2.02	2.02	2.11	33	33	367	9	37	1.32	2.13	3.17	12.2	99.46
	DODAB	2.04	2.07	2.14	60	18	260	8	39	1.14	2.14	3.18	11.9	99.91

**Table 3:** Kinect Parameters for Reaction of H_2_O_2_ with HRP and Fe(III)MP-11.

	HRP			Fe(III)MP-11		
	*K*_cat_ (s^−1^)	*K*_m_ (μM)	*K*_cat_/*K*_m_ (μM^−1^·s^−1^)	*K*_cat_ (s^−1^)	*K*_m_ (μM)	*K*_cat_/*K*_m_ (μM^−1^·s^−1^)
**HEPES**	10.53	109.90 ± 11.0	0.096	0.75	23.80 ± 2.2	0.031
**CTAB**	7.84	128.20 ± 5.4	0.061	1.00	71.43 ± 1.9	0.014
**DODAB**	8.88	151.51 ± 10.3	0.058	0.56	83.33 ± 5.71	0.0067
